# The Transcriptome Analysis of *Strongyloides stercoralis* L3i Larvae Reveals Targets for Intervention in a Neglected Disease

**DOI:** 10.1371/journal.pntd.0001513

**Published:** 2012-02-28

**Authors:** Antonio Marcilla, Gagan Garg, Dolores Bernal, Shoba Ranganathan, Javier Forment, Javier Ortiz, Carla Muñoz-Antolí, M. Victoria Dominguez, Laia Pedrola, Juan Martinez-Blanch, Javier Sotillo, Maria Trelis, Rafael Toledo, J. Guillermo Esteban

**Affiliations:** 1 Área de Parasitología, Departamento de Biologia Celular y Parasitología, Universitat de València, Burjassot, Valencia, Spain; 2 Department of Chemistry and Biomolecular Sciences, Macquarie University, Sydney, New South Wales, Australia; 3 Departamento de Bioquímica y Biología Molecular, Universitat de València, Burjassot, Valencia, Spain; 4 Department of Biochemistry, Yong Loo Lin School of Medicine, National University of Singapore, Singapore, Singapore; 5 Servicio de Bioinformática, Instituto de Biologia Molecular y Celular de Plantas, Universitat Politécnica de València, Ingeniero Fausto Elio, Valencia, Spain; 6 Unidad de Bioinformática, SCSIE, Universitat de València, Burjassot, Valencia, Spain; 7 Servicio de Microbiología y Parasitología, Hospital General de Castellón, Castellón, Spain; 8 LifeSequencing S.L., Parc Científic Universitat de València, Paterna, Valencia, Spain; McGill University, Canada

## Abstract

**Background:**

Strongyloidiasis is one of the most neglected diseases distributed worldwide with endemic areas in developed countries, where chronic infections are life threatening. Despite its impact, very little is known about the molecular biology of the parasite involved and its interplay with its hosts. Next generation sequencing technologies now provide unique opportunities to rapidly address these questions.

**Principal Findings:**

Here we present the first transcriptome of the third larval stage of *S. stercoralis* using 454 sequencing coupled with semi-automated bioinformatic analyses. 253,266 raw sequence reads were assembled into 11,250 contiguous sequences, most of which were novel. 8037 putative proteins were characterized based on homology, gene ontology and/or biochemical pathways. Comparison of the transcriptome of *S. strongyloides* with those of other nematodes, including *S. ratti*, revealed similarities in transcription of molecules inferred to have key roles in parasite-host interactions. Enzymatic proteins, like kinases and proteases, were abundant. 1213 putative excretory/secretory proteins were compiled using a new pipeline which included non-classical secretory proteins. Potential drug targets were also identified.

**Conclusions:**

Overall, the present dataset should provide a solid foundation for future fundamental genomic, proteomic and metabolomic explorations of *S. stercoralis*, as well as a basis for applied outcomes, such as the development of novel methods of intervention against this neglected parasite.

## Author Summary

*Strongyloides stercoralis* (Nematoda) is an important parasite of humans, causing Strongyloidiasis, considered as one of the most neglected diseases, affecting more than 100 million people worldwide. Chronic infections in endemic areas can be maintained for decades through the autoinfective cycle with the L3 filariform larvae. In these areas, misdiagnosis, inadequate treatment and the facilitation of hyperinfection syndrome by immunosupression are frequent and contribute to a high mortality rate. Among the affected areas, chronic patients have been described in the Valencian Mediterranean coastal region of Spain. Despite its serious impact, very little is known about this parasite and its relationship with its hosts at the molecular level, and more effective diagnostic tests and treatments are needed. Next generation sequencing technologies now provide unique opportunities to rapidly advance in these areas. In this study, we present the first transcriptome of *S. stercoralis* L3i using 454 sequencing followed by semi-automated bioinformatic analyses. Our study identifies 8037 putative proteins based on homology, gene ontology, and/or biochemical pathways, including putative excretory/secretory proteins as well as potential drug targets. The present dataset provides a useful resource and adds greatly to our understanding of a human parasite affecting both developed and developing countries.

## Introduction

Strongyloidiasis caused by *Strongyloides stercoralis* is a soil-transmitted helminthiasis distributed worldwide, affecting more than 100 million people, with endemic areas in Southeast Asia, Latin America, sub-Saharan Africa, and parts of the southeastern United States [1], [2]. Recently, it was classified as one of the most neglected tropical diseases (NTD) [3]. Chronic infections in endemic areas may be maintained asymptomatically for decades through the autoinfective cycle with the filariform larvae L3 [1] [4], [5]. The diagnosis of these chronic infections requires more sensitive diagnostic methods, particularly in low-level infections and immunocompromised patients [1].

Epidemiological studies in developed countries have identified endemic areas where misdiagnosis, inadequate treatment and the facilitation of hyperinfection syndrome by immunosupression (i.e. by the administration of steroids) are too frequent and can cause a high mortality rate ranging from 15 to 87% [5], [6]. Among these areas, an endemic area with chronic patients have been described at the Valencian Mediterranean coastal region of Spain related to environmental conditions [7].

The diagnosis of strongyloidiasis is suspected when clinical signs and symptoms, or eosinophilia is observed [8], but current definitive diagnosis of strongyloidiasis is usually made on the basis of detection of larvae in agar plate coproculture and serological diagnosis by ELISA [9], [10]. Those methods have the drawbacks of being time consuming and requiring expertise in the first case, and of low specificity due to remaining antibodies from previous infection or cross-reactive antibodies [11]. A recent paper has described a promising coproantigen ELISA based on a polyclonal rabbit antiserum raised against excretory/secretory (ES) antigens from the closely relative *Strongyloides ratti* [12], but the identification of *S. stercoralis* specific ES proteins that could be new potential targets for diagnosis is still required.

Control of strongyloidiasis has relied mostly on the treatment of infected individuals with only three anthelmintic drugs: thiabendazole (no longer available), albendazole, and more recently ivermectin [3], [13]. A recent study by Suputtamongkol *et al.* (2011) has confirmed that both a single and double dose of oral ivermectin are more effective than a 7-day course of high dose albendazole for patients with chronic infection due to *S. stercoralis* [14]. The risk of developing genetic resistance against the current drugs administered (if used excessively and at suboptimal dosages) exists and is based on the experience with drug resistance in parasitic nematodes of livestock [15]. Thus, the current focus is on the discovery of novel drugs against human parasites like *S. stercoralis*. Such a discovery effort could be strengthened with an integrated genomic and bioinformatics approach, using functional genomic and phenomic information available for the free-living nematode *Caenorhabditis elegans* (see WormBase; www.wormbase.org). This nematode, which is the best characterized metazoan organism [16], [17], is considered to be related to nematodes of the order Strongylida (to which *Strongyloides* belong) [18]. Recent studies have reported that nearly 60% of genes in strongyloides have orthologues/homologues in *C. elegans*, with a wide range of biological pathways being conserved between parasitic nematodes and *C. elegans* [19]. The comparison of molecular data sets between nematodes should therefore allow the identification of specific biological pathways as potential new targets for nematocidal drugs [20].

As pointed out recently by Cantacessi *et al.* (2011) [20], advances in genomic sequencing like Next Generation Sequencing (NGS) and annotation as well as the integrated use of ‘-omic’ technologies are now shedding light on our understanding of the systems biology of nematodes on an unprecedented scale, and is likely to provide unique opportunities for the development of entirely new strategies for the treatment and control of neglected parasitic diseases. New bioinformatic tools based on robust assembly protocol for NGS data, along with compilation of a dataset of experimentally determined ES proteins of parasitic helminths, and annotation software like KAAS [21], allow efficient and up-to-date homology-based predictions [22].

To date, there are few molecular and genomic studies on *Strongyloides* species, and only the transcriptome from *S. ratti* adults has become recently available (http://worm1.liv.ac.uk/file_summary.html) [23]. In fact, 39166 ESTs are currently available in the NCBI database of November 2011 (27366 from *S. ratti* and 11392 from *S. stercoralis*). Yoshida *et al.* (2011) have obtained 162 unique singletons and contigs from *S. venezuelensis* [24], and a recent study by Ramanathan *et al.* (2011) has described DNA microarray for *S. stercoralis* and used them to compare infective third-stage larvae (L3i) with non-infective first stage larvae (L1), with 935 differentially expressed genes identified [25].

In the present study, we have explored and functional annotated the transcriptome of L3i of *S. stercoralis* by 454 sequencing coupled to semi-automated bioinformatic analyses and predicted potential therapeutic targets for strongyloidiasis.

## Materials and Methods

### Accession numbers

The nucleotide sequence data obtained for this study are available in the GenBank database under accession number ERP000798.

The assembled data from this study can be requested from the corresponding author.

### Parasite material and ethical issues

Fecal samples were obtained at the Hospital La Ribera, Alzira, Valencia (Spain) from an infected individual in compliance with Spanish ethical regulations [7], and approved by the Ethics Committee in human research from the Universitat de Valencia. Oral consent from the patient was obtained (she was happy to participate in the study but felt uncomfortable with signing a form), and documented as a tick on the case record form following the Hospital Reviewing Board protocols. Samples were cultured on Agar Petri dishes and L3i larvae were harvested and concentrated by centrifugation for 5 min at 1000 g, washed three times in 1 ml of phosphate buffered saline (PBS) pH 7.2 containing protease inhibitors (10 mM EDTA, and 1 mM PMSF) and samples were processed for RNA isolation.

### RNA isolation, cDNA synthesis and 454 sequencing

Total RNA from around 500 larvae was prepared using Vantage™ Total RNA purification kit (Marligen Biosciences, Ijamsville, MD, USA) following the manufacturers' instructions and treated with Ambion DNA-free™ DNase (Ambion/Applied Biosystems, Austin, TX). The integrity of the RNA was verified by gel electrophoresis and the yield determined using the nanoDrop ND-1000 UV-VIS spectrophotometer v.3.2.1 (NanoDrop Technologies, Wilmington, DE).

The cDNA library was constructed from 0.5 µg total RNA using MINT cDNA Synthesis Kit (Cat#SK001, Evrogen). First strand cDNA synthesis starts from 3′-primer comprising oligo(dT) to enrich mRNA as template. Double strand cDNA synthesis was performed using 17 cycles of PCR amplification. Total cDNA was digested with restriction enzyme *GsuI* in order to remove Poly (A) tails. cDNA obtained was used to perform a library with the required sequencing adaptors and was then sequenced using the Genome Sequencer (GS) FLX instrument (Roche Diagnostics) [26].

### Bioinformatic analyses of sequence data

The overall bioinformatics analysis strategy followed was as described originally by Nagaraj *et al.* [27], [28], implemented in the analysis pipelines ESTExplorer [29] and EST2Secretome [28]. This workflow approach has been successfully used for the analysis of transcriptomic data from *Dictyocaulus viviparus* [30], *Fasciola hepatica* [31], *Clonorchis sinensis* [32] and *Opisthorchis viverrini* [32]. However, to better identify non-classically secreted proteins from helminth parasites [33], [34], we have recently implemented a novel analysis strategy for short reads applied on *Strongyloides ratti* [22] (see Figure S1).

FASTA and associated quality files were extracted from the SFF file after removing the sequence adapters. These reads were preprocessed and their contigs were assembled using MIRA v.3.2 (http://chevreux.org/projects_mira.html) [35] with the following parameters:

-job = denovo,est,accurate,454 -fasta

-OUT:rrol = 1:rld = 1:orc = 1:org = 0:ora = 0:ors = 0:otf = 0:otc = 0 -GE:not = 1 -CO:asir = 1

-LR:mxti = 1 -AS:sd = 0:uess = 0:urd = 0:ard = 1 -SK:mmhr = 2:mnr = yes 454_SETTINGS

-DP:ure = 0 -CO:mrpg = 10 -AS:bdq = 40

-CL:pvlc = 0:cpat = 0:mbc = 1:mbcgs = 30:mbcmfg = 30:mbcmeg = 30:qc = 0 -ED:ace = 1

-AL:egp = no

-ALIGN:bip = 20:bmax = 120:mo = 10.

Contigs generated from MIRA were aligned and reassembled into second order contigs using the Contig Assembly Program v.3 (CAP3) [36], employing a minimum sequence overlap length cut-off of 40 bases and an identity threshold of 90%. Following the assembly of *S. stercoralis* reads into second order contigs by CAP3 and contigs by MIRA, this contig dataset was matched using BLASTX with the NCBI non-redundant sequence database; http://www.ncbi.nlm.nih.gov, BLASTN with Nematode.net *S. stercoralis* ESTs (www.nematode.net/) and BLASTN with dbEST *Strongyloides* ESTs (www.ncbi.nlm.nih.gov/dbEST/), using permissive (E-value: <1E^−05^), moderate (<1E^−15^) and/or stringent (<1E^−30^) search strategies.

*S. stercoralis* contigs were conceptually translated into putative proteins using the program ESTScan [37]. Putative protein sequences were subjected to secretome analysis using TMHMM (a membrane topology prediction program) [38] to predict transmembrane domains, SignalP 3.0 (signal peptide prediction program) [39], SecretomeP (a prediction programme used to identify non-classical secretory proteins in mammals [40], but used in the case of parasitic helminths as well [41]), and TargetP (mitochondrial protein prediction program) [42]. Briefly, excretory/secretory (ES) proteins were selected based on the presence of a signal peptide at the N-terminus using SignalP 3.0 (employing both the neural network and hidden Markov models) or predicted as secretory using SecretomeP, predicted as non-mitochondrial by TargetP and absence of transmembrane domains. In addition to computational prediction of ES proteins were identified and collated based on sequence homology (BLASTP, E-value<1E^−15^) to known ES proteins found in parasitic helminths secretome studies.

Putative proteins were classified functionally using InterProScan [43], employing the default search parameters. Based on their homology to conserved domains and protein families, predicted proteins were classified into Gene Ontology (GO) categories (http://www.geneontology.org/) based on molecular function, cellular component and biological process using interpro terms. Putative proteins were also subjected to pathway analysis, utilizing KEGG-Automatic Annotation Server (KAAS) [21], which maps the putative proteins to biochemical pathways in which they are involved and categories of Brite objects like enzymes, transcription factors and translation factors.

Putative proteins were subjected to BLAST2GO software to identify homologues from the most abundant ES transcripts [44]. BLASTP (Wormpep v 224) was used to identify *C. elegans* known proteins homologues present in *S. stercoralis* proteins using moderate search strategy (E-value: <1E^−15^). These proteins were also searched for sequence homology (BLASTP, E-value<1E^−05^) in human (host) proteins. All the proteins which were found homologous to *C. elegans* proteins and non-homologous to human proteins were mapped to *C. elegans* RNAi phenotypes and known drug targets present in the DrugBank database (http://drugbank.ca/), a unique bioinformatics and cheminformatics resource that combines detailed drug (i.e. chemical, pharmacological and pharmaceutical) data with comprehensive drug target (i.e. sequence, structure, and pathway) information [45].

## Results

### The transcriptome of *S. stercoralis* L3i larvae

Initially a total of 253266 short reads (82490223 bases) were generated with 325±132.4 bases (average length ± standard deviation), with a GC content of 31.84%. These short reads were pre-processed, which resulted in 237341 (93.7%) quality short reads (EBI Sequence Read Archive [SRA] accession ID ERP000798). High quality reads were assembled into 12333 contigs using MIRA as described in the pipeline (Figure S1). Using CAP3, we were able to achieve 507 second order contigs, leaving 10845 MIRA contigs not assembled further by CAP3. We considered 11250 (99.1%) contigs with a minimum length of 90 bases, discarding sequences yielding peptides <30 amino acids, for further secretory protein prediction and analysis. These contigs were conceptually translated into 8037 proteins by ESTScan (Table 1; sequences available from http://biolinux.uv.es/marcilla/).

**Table 1:** Expressed sequence tag (EST) data for the *S. stercoralis* L3i.

No. of EST Clusters by MIRA	12,333
Average length (±standard deviation)	538.7 (±260.7)
Re clustered unigenes after CAP3 assembly	11,352
Containing an Open Reading Frame	8,037
Returning InterProScan results	4,494
Gene Ontology	3,534
Biological process	1,905
Cellular component	1,068
Molecular function	3,083
Prediction of biological pathways (KAAS)	1,559

Putative proteins were annotated based on protein families and domains using Interproscan and mapped to biochemical pathways using KAAS [21]. Of the 8037 putative proteins, we were able to annotate 4494 (55.91%) proteins with protein domains and families (Table 1). The most represented Interpro terms are shown in Table S1.

A total of 3534 proteins were annotated with GO terms (3083 {Molecular Function}, 1068 {Cellular Component} and 1905 {Biological Process}) based on Interpro term annotations (Tables 1 and S1). We established pathway associations for 1559 (19.39%) putative proteins (Table 1).

All the contigs generated by using MIRA+CAP3 were checked for homologous proteins against the non-redundant nucleotide database (NR-NCBI), existing *Strongyloides* expressed sequence tags (ESTs) present in dbEST, *S. stercoralis* ESTs available from dbEST and nematode.net, *S. ratti* cDNA sequencing data from the University of Liverpool (available at http://worm1.liv.ac.uk/file_summary.html), and also for homologous proteins in *C. elegans* and human data (Figure S1). Similarity searches were done using using BlastX and BlastP algorithms at different E values (Table 2). A total of 3412 (42.45%) *S. stercoralis* putative proteins had homologues in the free-living nematode, *Caenorhabditis elegans* using stringent match conditions (E value: <1E^−15^). The recent availability of *S. ratti* transcriptome data prompted us to compare these with our data and 3855 similar putative proteins (47.96%) were found. As *S. stercoralis* infects humans, we checked the similarity of *S. stercoralis* proteins with known human proteins using BlastP at different E values. Our results showed that 3759 putative proteins were similar to human ones using a permissive search strategy (E-value: <1E^−05^), discarding them as potential targets for treatment (Table 2).

**Table 2:** Sequence homology inferred between *S. stercoralis* current dataset and other datasets.

Dataset	Hits (1e-05)	Hits (1e-15)	Hits (1e-30)
*Strongyloides* EST from dbEST (BLASTN)	4267	3935	3646
*S. stercoralis* EST from dbEST (BLASTN)	2903	2682	2519
*S. stercoralis* EST from nematode.net (BLASTN)	2225	2020	1904
*S. ratti* putative proteins (BLASTP)	3855	3052	2081
*C. elegans* proteins (BLASTP)	4475	3,412	2163
Human proteins (BLASTP)	3759	2583	1489
NR database (BLASTX)	7633	5948	4023

Predicted proteins were also categorized according to their inferred molecular function, cellular localization and association with biological pathways. Mapping to KEGG BRITE objects [46] is shown in Table 3. Enzymes were by far the most abundant category, with 720 putative proteins, followed by chromosome, spliceosome and ribosome components (with 90, 89 and 73 putative proteins, respectively). 73 putative protein kinases and 72 peptidases were also identified by BRITE (Table 3). These 72 peptidases corresponded to 60 different enzymes from 9 groups, including calpains, cathepsins, different proteasome components and aminopeptidases, and other “nematode common” proteases such as astacin, legumain, and insulysin (Table S2).

**Table 3:** Functions of putative proteins inferred from the transcriptome of the *S. stercoralis* L3i.

BRITE object name	Number of putative proteins mapped
Enzymes	720
Chromosome	90
Spliceosome	89
Ribosome biogenesis	73
Protein kinases	73
Peptidases	72
Ubiquitin system	72
Ribosome	72
Chaperones and folding catalysts	61
Cytoskeleton proteins	53
Proteasome	41
Ion Channels	38
Transcription factors	36
Translation factors	34
DNA repair and recombination proteins	32
GTP-binding proteins	25
DNA replication proteins	20
Glycosyltransferases	15
Lipid biosynthesis proteins	14
Cellular antigens	11
Transporters	11
Secretion system proteins	10
Glycan binding proteins	9
SNAREs	7
Nuclear receptors	6
Prenyltransferases	6
G protein coupled receptors	4
CAM ligands	4
Proteoglycans	4
Enzyme linked receptors	2
Cytokine receptors	2
Cell adhesion molecules (CAMs)	2
Bacterial toxins	1

All the putative proteins were grouped according to KEGG pathways [46] into five categories, with metabolic proteins being the most abundant, followed by genetic information processing, environmental processing and cellular processes (Table 4). In the first group, the most abundant putative proteins were related to carbohydrate metabolism (201 proteins, 2.5%), amino acid metabolism (174; 2.16%) and lipid metabolism (104; 1.29%). Also 23 putative proteins were related to drug metabolism (Table 4). In the second group, the most abundant proteins were related to translation (195; 2.42%), meanwhile 144 putative proteins (1.79%) related to signal transduction were the most abundant in the group of cellular processes (Table 4).

**Table 4:** KEGG pathways of putative proteins inferred from the transcriptome of *S. stercoralis* L3i.

Parent KEGG pathway	No of putative proteins (%)	Top KEGG pathway in the category
**Metabolism**		
Carbohydrate metabolism	201 (2.50)	Glycolysis/Gluconeogenesis
Energy metabolism	79 (0.98)	Oxidative phosphorylation
Lipid metabolism	104 (1.29)	Fatty acid metabolism
Nucleotide metabolism	100 (1.24)	Purine metabolism
Amino acid metabolism	174 (2.16)	Valine, leucine and isoleucine degradation
Metabolism of other amino acids	48 (0.60)	Glutathione metabolism
Glycan biosynthesis and metabolism	30 (0.37)	N-Glycan biosynthesis
Metabolism of Cofactors and Vitamins	46 (0.57)	One carbon pool by folate
Metabolism of Terpenoids and Polyketides	5	Terpenoid backbone biosynthesis
Biosynthesis of other secondary metabolites	1	Caffeine metabolism
Xenobiotic biodegradation and metabolism	23 (0.29)	Drug metabolism – other enzymes
**Genetic Information processing**		
Transcription	83 (1.03)	Spliceosome
Translation	195 (2.42)	Ribosome
Folding, sorting and degradation	178 (2.21)	Protein processing in endoplasmic reticulum
Replication and repair	34 (0.42)	Nucleotide excision repair
**Environmental information processing**		
Membrane transport	3	ABC transporters
Signal transduction	144 (1.79)	MAPK signaling pathway
Signaling, molecules and interaction	7	Neuroactive ligand-receptor interaction
**Cellular processes**		
Transport and catabolism	132 (1.64)	Endocytosis
**Organism systems**		
Immune system	7	Natural killer cell mediated cytotoxicity
Endocrine system	11 (0.13)	Progesterone-mediated oocyte maturation
Development	3	Dorso-ventral axis formation
Environmental adaptation	6	Circadian rhythm – mammal

### Prediction of ES proteins

We next analyzed ES proteins, which are key molecules to understand host-parasite interactions [47]. Molecules from the secretome contribute to important processes like parasite feeding, tissue penetration or larval migration, and could participate in blocking and/or evading host immune responses [48]. ES prediction was carried out in Phase III of the pipeline (Fig. S1). Firstly, 247 (3.07%) proteins were predicted as classical secreted proteins using SignalP [39]. The remaining 7785 (96.86%) proteins, which were predicted as non-secretory by SignalP were processed by SecretomeP [40] for prediction of non-classical secretory proteins, with 252 (3.14%) proteins identified here. The classical and non-classical secretory proteins (499, 6.21%) from these two programs were analyzed by TargetP [42] for mitochondrial proteins. Only 7 proteins were predicted as mitochondrial proteins using TargetP at 95% specificity. These seven proteins were removed from the set of 499 secreted proteins, with 492 secretory proteins passed to TMHMM [38] for the prediction of transmembrane proteins. 161 (2%) proteins, were predicted as transmembrane proteins having one or more transmembrane helices, and removed from the secretory protein dataset. A total of 331 (4.12%) proteins were finally predicted as ES proteins from the computational prediction pipeline. Proteins that were considered non-secretory by SecretomeP and SignalP were matched to our in-house dataset of 1080 non redundant experimentally determined parasitic helminth proteins [22] using the BLASTP [49] similarity search. We found an additional 882 (10.97%) putative proteins similar to known ES proteins by this homology search approach (E value: <1E^−15^) (Table S3). From those proteins, 50 have been recently described in the ES from infective larvae of the related species *S. ratti* [50] (data not shown).

Among the most abundant transcripts encoding ES proteins appeared a major antigen; cytoskeletal proteins like myosin heavy chain, troponin, tropomyosin, actin; galectins; enzymes like trehalase, PEPCK, GAPDH, enolase, as well as phosphatases and kinases; proteases like Metalloproteinase, Calpain-1and Cathepsin L; stress proteins like HSPs; calcium binding proteins; detoxifying enzymes along with elongation factors, histones, ubiquitins and signaling molecules (Table S4. Thus, for annotation and analyses in Phase III, we compiled a total of 1213 ES proteins, which is 15.09% of our putative proteins.

### *S. stercoralis* proteins as drug targets

We found 4234 (52.68%) *S. stercoralis* putative proteins which had no homologues present in humans (Table 2) and therefore are preferred targets for parasite intervention strategies. These human dissimilar proteins of *S. stercoralis* were checked for known drug targets, which have lethal RNAi phenotypes present in *C. elegans*, not present in human and similar to known drug targets, data available from DrugBank 3.0 database [44], a unique bioinformatics and cheminformatics resource that combines detailed drug (i.e. chemical, pharmacological and pharmaceutical) data with comprehensive drug target (i.e. sequence, structure, and pathway) information. The database (available at http://drugbank.ca/) contains 6707 drug entries (as of November 2011).

We found 14 contigs and singletons corresponding to four different proteins. These could represent potential therapeutic targets for strongyloidiasis as shown in Table 5. Sequence comparison demonstrate that these proteins are homologous to 2,3-bisphosphoglycerate independent phosphoglycerate mutase from *Ascaris suum* (with 1 contig and 1 singleton), hypothetical protein CBG01975 from *Caenorhabditis briggsae* similar to glutamate synthase [NADPH] from *Ascaris suum* (1 contig and 3 singletons), isocitrate lyase from *S. stercoralis* (5 singletons), and alcohol dehydrogenase I from the fungus *Candida albicans* WO-1 (2 singletons) (Table 5).

**Table 5:** Predicted therapeutic targets from *S. stercoralis* L3i.

No	Cluster ID	Description from NR Database	WBGene ID	*C. elegans* RNAi lethal phenotype	Interpro hits	*C. elegans* Gene Ontology	Drugbank targets hits	ESP
1	Contig 19 Singleton 1215	2,3-Bisphosphoglycerate independent phosphoglycerate mutase (*Ascaris suum*)	WBGene 00019001	Slow growth; embryonic lethal; larval arrest; organism morphology abnormal; egg laying abnormal; locomotion abnormal; life span abnormal	IPR006124 IPR017850	BIOLOGICAL PROCESSES: glucose catabolic process; positive regulation of growth rate; embryonic development ending in birth or egg hatching; nematode larval development; body morphogenesis; locomotion; determination of adult life span; oviposition. MOLECULAR FUNCTION: phosphoglycerate mutase CELLULAR COMPONENT: cytoplasm	2,3-Bisphosphoglycerate independent phosphoglycerate mutase	yes
2	Contig 462 Singletons 600, 2555, 3841	Hypothetical protein CBG01975 (*Caenorhabditis briggsae*)Glutamate synthase [NADPH] (*Ascaris suum*)	WBGene 00012326	Slow growth; embryonic lethal; larval arrest	IPR000583 IPR002932 IPR017932 IPR002489	BIOLOGICAL PROCESSES: electron transport; glutamate biosynthetic process; nitrogen compound metabolic process; embryonic development ending in birth or egg hatching; nematode larval development; positive regulation of growth rate. MOLECULAR FUNCTION: oxidoreductase activity, acting on the CH-NH2 group of donors, NAD or NADP as acceptor FAD binding; glutamate synthase activity, NADH or NADPH as acceptor; iron-sulfur cluster binding	Ferredoxin-dependent glutamate synthase 2	No
3	Singletons 77, 154, 179, 2466, 10457	Isocitrate lyase (*Strongyloides stercoralis*)	WBGene 00001564	Life span abnormal	IPR000918 IPR006254 IPR015813	BIOLOGICAL PROCESSES: glyoxylate cycle; carboxylic acid metabolic process; embryonic development; determination of adult life span. MOLECULAR FUNCTION: isocitrate lyase activity; malate synthase activity	Isocitrate lyase	No
4	Singletons 2046, 10787	Alcohol dehydrogenase I (*Candida albicans* WO-1)	WBGene 00010790	Life span abnormal	IPR002085 IPR011032 IPR013149	BIOLOGICAL PROCESSES: metabolic process determination of adult life span MOLECULAR FUNCTION: zinc ion binding oxidoreductase activity	Alcohol dehydrogenase	No

Putative proteins homologous to *C. elegans* proteins with lethal RNAi phenotype and with no homologue in the human host.

With a comparative analysis searching protein domain mapping or sequence similarity with other drug targets, we found seven additional potential targets for treatment, including well known drug targets as tubulin β, γ-amino butyric acid A (GABA) receptor, glutamate-gated chloride channel or GST (Table S5). Only one of those proteins, homologous to *Ancylostoma caninum* metalloprotease precursor, which is also predicted to be secretory, was not found similar either to *C. elegans* or human proteins (Table S5).

## Discussion

*Strongyloides stercoralis* can replicate within the host (autoinfection) allowing the infection to remain undiagnosed and untreated for years, resulting in perpetuating parasite dispersal, increasing the risk of infection and eventually the appearance of resistances [3]. Uncontrolled multiplication of the parasite (hyperinfection) can be life-threatening in immunocompromised individuals. We also face serious endemic recurring infections in the future if this infection is not controlled in transition economies like China, India, Southeast Asia and Latin America [3] where the use of immunosuppressive therapy is becoming common. As pointed out by Olsen *et al.* (2009) [3], there is an urgent need to employ modern molecular methods to improve and simplify diagnosis, differentiate species and strains to facilitate epidemiological studies of *S. stercoralis*.

The present study provides the first detailed analysis of the transcriptome of the human pathogenic *S. stercoralis* L3i larvae and has identified specific molecules predicted to play key biological functions in this parasite. A total of 12,333 contigs were inferred from the present EST dataset, thus increasing the number of predicted proteins currently available (for this stage/species) in public databases by approximately 141-fold [we obtained 8037 conceptually translated proteins, and there are currently 57 proteins in Genbank as of November 2011]. This quantity of contigs is similar to the numbers obtained with other nematodes like *Trichostrongylus colubriformis* [19], *N. americanus* and *Ancylostoma caninum* [51], *Haemonchus contortus* [52], *Dictyocaulus viviparous* [20], and *Teladorsagia circumcinta* [53]. The subset (55.91%) of *S. stercoralis* sequences with orthologues/homologues in public databases was slightly higher to that reported in similar transcriptomic studies of other animal-parasitic helminths such as *Necator americanus* [51], [54]. It is noteworthy to mention that 44.09% of the putative proteins of *S. stercoralis* L3i transcriptome remain unannotated, warranting further genomic and functional characterization studies.

With the exception of three metabolic proteins (citrate synthase, arginine kinase and ATP:guanido phosphotransferase) all proteins identified in a previous proteomic study with *S. stercoralis* L3i [55] were included in the transcriptome described here. In addition, 41 antigenic proteins including SiR and tropomyosin were present in the transcriptome (as searched in Table S1), confirming its value as a tool for searching targets for immunodiagnosis.

It is well characterized that upon infection, infective larvae (L3i) must penetrate skin as quickly as possible and then migrate within the host. In this context, proteases play an essential role. Among the proteins identified in our study, 60 different putative proteases were annotated in nine groups. These include nine metalloproteinases and three aspartic proteases, some of them assumed to play a major role in skin penetration in *Strongyloides stercoralis* [56], [57], and in other *Strongyloides* species like *S. venezuelensis* [58] or *S. ratti* [22], [23]. In *S. venezuelensis*, Yoshida *et al.* (2011) [24] have recently identified an astacin-like metalloproteinase as being specific of L3i in a transcriptomic study. Another abundant group was the cysteine proteases, including cathepsin B, legumain and calpain, proteins characterized as immunomodulators of host response and promising vaccine and drug targets [59]–[61]. Similar results have been reported for *Ascaris suum*, where 456 peptidases have been identified in its draft genome [62].

Kinases are also an important group of proteins considered to be good druggable targets from the medical and chemical viewpoints, since they play essential functions in the parasite, in mediating signal transduction [63]–[65]. In *S. stercoralis* L3i transcriptome analysis 73 putative kinases including 11 putative tyrosine kinases were identified (Table 3 and Table S1).

In our study, we have compiled 1213 putative ES proteins among the 8037 (15.09%) *S. stercoralis* annotated proteins using a new semi-automated computational approach, recently developed and applied to predict the secretome of *S. ratti* adults [22]. In a mixture of *S. ratti* parasitic females, free-living males and free-living females, Garg and Ranganathan (2011) compiled 2572 putative ES proteins, being 12.3% of the total putative proteins, which is less than that found in *S. stercoralis* L3i larvae [22]. This could be due to higher secretion processes in larvae in comparison to adults, required by penetration and migration in the host. Supporting this notion, Soblik et al. (in press) have recently described the presence of 586 ES proteins in all the stages of *S. ratti* by proteomic analysis, 196 of which are also found in L3i [50]. When comparing larval *S. ratti* ES proteins with our predicted *S. stercoralis* L3i ES proteins, we find that 50 out of the 196 proteins identified from *S. ratti* were also detected in *S. stercoralis* L3i, supporting the value of the prediction.

In *S. stercoralis* L3i, the most abundant transcripts encoding ES proteins include cytoskeletal proteins (i.e. myosin heavy chain, actin, tropomyosin, tubulin or paramyosin), metabolic enzymes (i.e. Trehalase, PEPCK, PGK, PGM, GAPDH, enolase), proteases, stress-response proteins, detoxifying enzymes, proteaseome components, most of them identified previously in *S. stercoralis* by proteomic studies [55]. These ES proteins play a major role in infection since they are present at the host-parasite interface and regulate host immune system [66]. ESPs also are among the target choice of new therapeutic solutions for helminth infections [67], as confirmed in the case of ivermectin (the currently the drug of choice for treating strongyloidosis) which has been shown to act reducing the secretion of ESPs from the ES apparatus in *Brugia malayi* microfilariae [68].

Recent studies using microarrays have identified highly expressed molecules in *S. stercoralis* L3i in comparison to L1 larvae, including cytochrome bc1, Hsp-90 and FAR-1, which potentially constitute new targets for intervention [25], all of which were present in our transcriptome data, but did not appear as druggable targets following our pipeline, possibly as these are not present in DrugBank, where only lethal RNAi phenotypes are included. Other important targets if interfered with, would still lead to expulsion of live worms form a host, like motility genes. In agreement with this, Garg and Ranganathan (2011) [22] have recently identified 19 contigs as putative drug targets in the *S. ratti* adult transcriptome, including myosin heavy chain, which is also one of the most abundant transcript of ES proteins in *S. stercoralis* (Table S4). This protein along with others like a metalloproteinase precursor, major sperm protein or triosephosphate isomerase (also identified in the *S. stercoralis* transcriptome in our study) did not appear as druggable molecules in our study, due to the presence of these proteins in host cells as well. In this context, efficient drugs as antihelmintics like benzimidazoles (they inhibit tubulin ß resulting in impaired microtubule formation during cell division) have much more affinity for tubulin in helminth cells than the tubulin found in the cells of mammals [69]. We found 11 potential targets for treatment against L3i larvae. As already mentioned, these are the first evolutive phase of *S. stercoralis* in the host, and constitute a good target for treatment. From these target molecules, four, with no homologues in the host, suggesting parasite specificity, are: 2,3-bisphosphoglycerate independent phosphoglycerate mutase, glutamate synthase, isocitrate lyase and alcohol dehydrogenase I. Only the first one was predicted as present in ES. Further studies are required to confirm whether these molecules are good drug targets for strongyloidiasis. Next-generation sequencing technologies are improving genomic and transcriptomic studies, and complemented by proteomic investigations, should allow the characterization of differential gene expression and essential pathways in all the developmental stages of *S. stercoralis*. The transcriptomic dataset described here constitutes the basis for future investigations enlightening the search for control measures for one of the most neglected diseases.

## Supporting Information

Figure S1 **Bioinformatics workflow used for transcriptomic data analysis.** Bioinformatics workflow comprising Phase I (pre-processing and assembly), II (Nucleotide level annotation), III (prediction of excretory/secretory (ES) proteins) and IV (Protein-level annotation).
(DOC)

Table S1 ***Strongyloides stercoralis*** **L3i gene ontology mapping using Interproscan.**
(XLS)

Table S2 **Peptidases found in the** ***S. stercoralis*** **L3i transcriptome.**
(XLS)

Table S3 **BLAST results of proteins found homologous to experimentally verified secretory proteins of parasitic helminths at 1e-^15^.**
(XLS)

Table S4 **Top 50 abundant transcripts across** ***Strongyloides stercoralis*** **L3i excretory/secretory proteins using Blast2Go** [**44**] **.**
(DOCX)

Table S5 ***S. stercoralis*** **L3i putative proteins found similar to known therapeutic targets in parasitic nematodes either by protein domains mapping or sequence similarity.**
(DOCX)
