# Supplementary figures and images for: The Transcriptome Analysis of Strongyloides stercoralis L3i Larvae Reveals Targets for Intervention in a Neglected Disease
Source: PLoS Negl Trop Dis. 2012 Feb 28;6(2):e1513. doi: 10.1371/journal.pntd.0001513 (PMC3289599; doi:10.1371/journal.pntd.0001513)

**
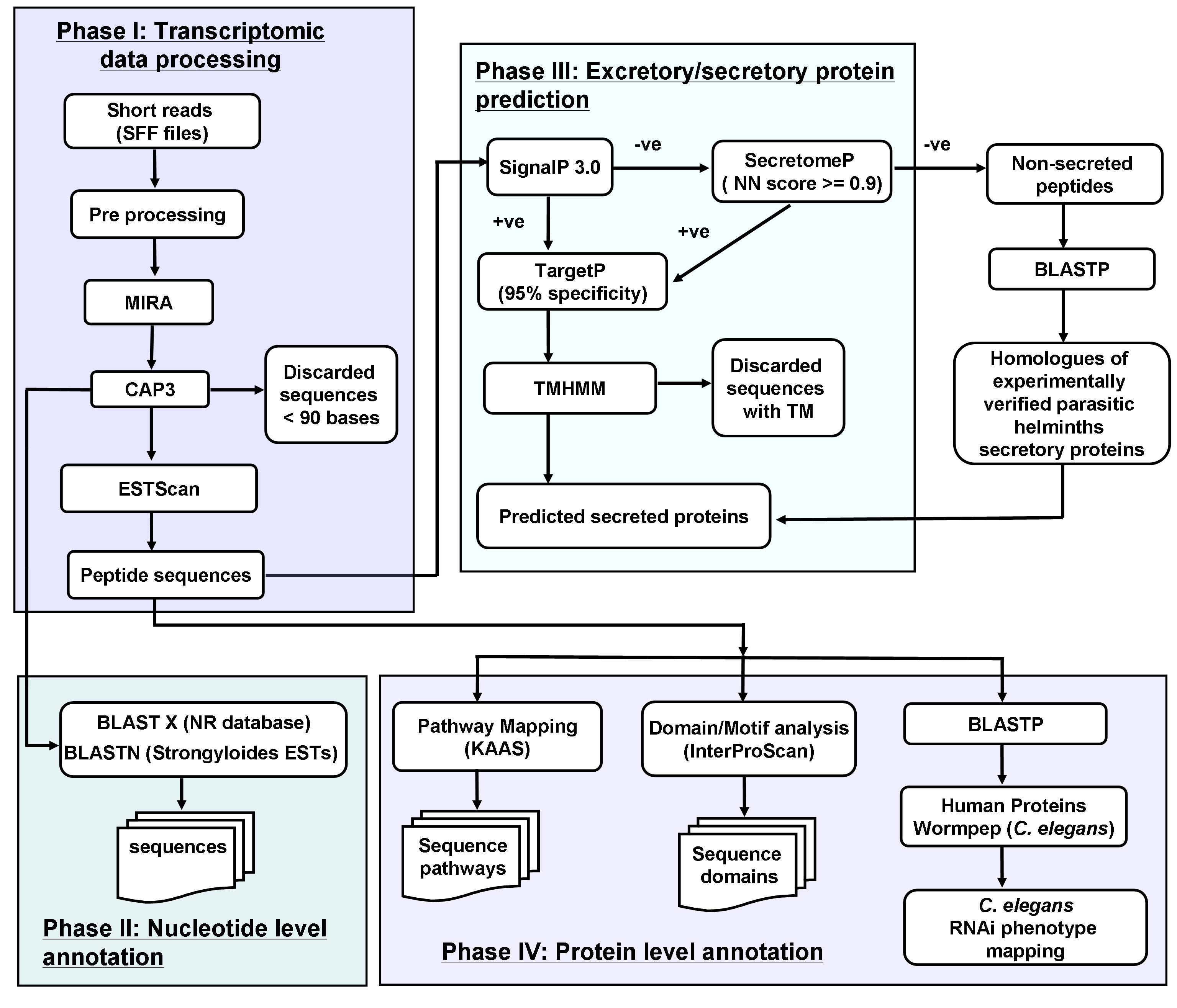
**

Supplement: Figure S1 — Bioinformatics workflow used for transcriptomic data analysis. Bioinformatics workflow comprising Phase I (pre-processing and assembly), II (Nucleotide level annotation), III (prediction of excretory/secretory (ES) proteins) and IV (Protein-level annotation). (DOC) [file pntd.0001513.s001.doc]
