# Supplementary material for: The Transcriptome Analysis of Strongyloides stercoralis L3i Larvae Reveals Targets for Intervention in a Neglected Disease
Source: PLoS Negl Trop Dis. 2012 Feb 28;6(2):e1513. doi: 10.1371/journal.pntd.0001513 (PMC3289599; doi:10.1371/journal.pntd.0001513)
Supplement: Table S4 — Top 50 abundant transcripts across Strongyloides stercoralis L3i excretory/secretory proteins using Blast2Go [44] . (DOCX) [file pntd.0001513.s005.docx]

**Table S4. Top 50 abundant transcripts across *Strongyloides stercoralis* L3i excretory/secretory proteins using Blast2Go [44]**

| **No** | **Query ID** | **No of reads** | **Sequence Description** | **Species** | **Accession No** | **min e value*** | **% similarity*** |
| --- | --- | --- | --- | --- | --- | --- | --- |
| 1 | Singleton2, contig19 | 1849 | Major antigen | *Ascaris suum* | ADY39937 | 4,96E-28 | 76.0 |
| 2 | Singletons13,174, 1297,1481,2068,3351,5188,7523, 5451, 5489,8115; contigs52,276,389,492 | 1714 | Myosin heavy chain B | *Caenorhabditis elegans* | P02566 | 7,06E-37 | 92.0 |
| 3 | Contig3 | 1680 | Trehalase | *Ascaris suum* | ADY41420 | 7,88E-131 | 70.0 |
| 4 | Contig12, singleton5 | 1024 | Troponin T | *Ascaris suum* | ADY44986 | 8,90E-54 | 76.0 |
| 5 | Contig8, singletons608,1562 | 850 | Metalloproteinase precursor | *Strongyloides stercoralis* | AAK55800 | 4,84E-33 | 100.0 |
| 6 | Singletons8,102, 813, 1298, 9079, 8848 | 594 | Calpain1 | *Caenorhabditis elegans* | NP_498740 | 6,71E-96 | 84.0 |
| 7 | Singleton28, contig53 | 520 | Tropomyosin-2 | *Bursaphelenchus xylophilus* | ACZ13334 | 8,14E-43 | 97.0 |
| 8 | Singletons14,6361,2792, | 512 | Phosphoenolpyruvate carboxykinase (PEPCK) | *Ascaris suum* | ADY42920 | 7,12E-23 | 84.0 |
| 9 | Singletons39,2792,2367,2487,7706, contig49 | 471 | 14-3-3 protein | *Ascaris suum* | ADY42720 | 6,29E-84 | 94.2 |
| 10 | Contig24, singletons120,390 | 444 | Galectin | *Teladorsagia circumcincta* | AAC47546 | 6,59E-46 | 89.0 |
| 11 | Contig28, singletons503,2363,3531,3925,7939, 298, 704,1873,4531 | 432 | Glyceraldehyde-3-P dehydrogenase (GAPDH) | *Caenorhabditis remanei* | EGT30142 | 1,17E-69 | 93.2 |
| 12 | Contig52 | 393 | Hypothetical protein CRE_29635 | *Caenorhabditis remanei* | XP_003111956 | 3,41E-04 | 48.67 |
| 13 | Contig79 | 355 | Ubiquitin family protein | *Trichinella spiralis* | XP_003381631 | 5,02E-22 | 100.0 |
| 14 | Singleton20 | 352 | Cathepsin L | *Strongylus vulgaris* | ABW25047 | 4,32E-40 | 84.0 |
| 15 | Contig32 | 332 | Glutathione peroxidase | *Haemonchus contortus* | ACX55058 | 1,76E-49 | 82.0 |
| 16 | Singletons72,434, 164 | 325 | Copine family protein | *Ascaris suum* | ADY39806 | 4,84E-85 | 92.0 |
| 17 | Singleton80 | 310 | Puromycin-sensitive aminopeptidase | *Loa loa* | XP_003143809 | 8,49E-146 | 64.2 |
| 18 | Contig19 | 299 | Independent Phosphoglycerate mutase (PGM) isoform2 | *Ascaris suum* | ADY42070 | 8,65E-46 | 83.0 |
| 19 | Contig38, singleton848 | 296 | Hypothetical protein CAEBREN_25766 | *Caenorhabditis brenneri* | EGT59396 | 1,90E-29 | 46.7 |
| 20 | Singletons224,214,203,1547,4060,9813 | 279 | Actin | *Bursaphelenchus xylophilus* | BAI52957 | 0.0 | 99.0 |
| 21 | Singletons57,5472,6186,9654, 904, 1614, 4314 | 279 | Heat shock protein 70 (HSP-70) | *Onchocerca volvulus* | P11503 | 1,44E-52 | 96.0 |
| 22 | Singleton59 | 273 | Propionyl-coenzyme a alpha polypeptide | *Ascaris suum* | ADY41700 | 0 | 84.0 |
| 23 | Singleton65 | 245 | Helix loop helix family member (HLH-30) | *Brugia malayi* | XP_001893227 | 8,22E-32 | 61.0 |
| 24 | Singletons142,236,723,9558 | 245 | Heat shock protein 90 (HSP-90) | *Brugia pahangi* | CAA06694 | 0.0 | 89.0 |
| 25 | Contig27 | 241 | Ferritin-1 | *Caenorhabditis briggsae* | XP_002635429 | 2,87E+01 | 75.0 |
| 26 | Singleton29 | 238 | 3-Ketoacyl- mitochondrial-like | *Ascaris suum* | ADY45399 | 8,69E-68 | 71.7 |
| 27 | Singletons79,576 | 238 | Calmodulin | *Caenorhabditis elegans* | CAA10601 | 1,66E-62 | 99.7 |
| 28 | Singleton129 | 223 | Ser protein kinase family member (SPK-1) | *Caenorhabditis elegans* | NP_499080 | 1,76E-125 | 80.0 |
| 29 | Singletons121,245,450,6474 | 223 | Histone H2b | *Culex quinquefasciatus* | XP_001862657 | 8,93E-55 | 91.0 |
| 30 | Singletons207,299, 3894 | 217 | Tubulin alpha chain | *Ascaris suum* | ADY46322 | 1,17E-162 | 95.5 |
| 31 | Contig34 | 213 | Protein kinase C | *Ascaris suum* | ADY42447 | 1,16E-124 | 94.4 |
| 32 | Singletons56,7139 | 212 | Fumarase family member (FUM-1) | *Ascaris suum* | AAP51177 | 1,93E-95 | 92.0 |
| 33 | Singletons32,5036,10748 | 208 | Elongation factor 1-α | *Meyerozyma guilliermondii* | XP_001483189 | 2,29E-54 | 98.0 |
| 34 | Singletons215,551,478,1522,1554,2689,4694,8231 | 208 | Glutamine synthetase | *Caenorhabditis brenneri* | EGT42094 | 1,57E-143 | 86.0 |
| 35 | Singleton43 | 203 | Serine threonine-protein phosphatase PP1-α catalytic subunit | *Candida tropicalis* | XP_002551201 | 4,08E-115 | 85.8 |
| 36 | Contig31 | 176 | Calcium-transporting ATPase | *Caenorhabditis elegans* | NP_001122708 | 3,20E-78 | 90.0 |
| 37 | Singleton158 | 171 | Tropomodulin family protein | *Brugia malayi* | XP_001900977 | 2,99E-107 | 75.2 |
| 40 | Singletons99,461 | 171 | Aspartic protease SP-2 | *Strngyloides ratti* | ACR56788 | 2,33E-31 | 89.6 |
| 41 | Singleton50 | 169 | ATP synthase beta | *Bursaphelenchus xylophilus* | ACZ13324 | 3,75E-63 | 98.0 |
| 42 | Singletons182,2715,1807,4345,3158,3652,6176 | 155 | Trehalose 6-phosphate synthase | *Ascaris suum* | ADY40229 | 1,42E-79 | 72.0 |
| 43 | Singletons133,415,2963,8056 | 150 | Enolase | *Caenorhabditis elegans* | NP_871916 | 1,01E-65 | 94.0 |
| 45 | Singleton89 | 137 | Histone H3 | *Nematostella vectensis* | XP_001618471 | 1,57E-38 | 98.0 |
| 46 | Singleton333 | 137 | Paramyosin | *Onchocerca volvulus* | AAA29431 | 0.0 | 96.0 |
| 47 | Singletons238,314 | 136 | Serine threonine-protein phosphatase PP1-β catalytic subunit | *Brugia malayi* | XP_001894042 | 6,16E-72 | 89.0 |
| 48 | Contig56 | 133 | Ubiquitin-conjugating enzyme E2R 2 | *Ascaris suum* | ADY47034 | 7,35E-92 | 86.0 |
| 49 | Singleton111 | 126 | Amidinotransferase family protein | *Ascaris suum* | ADY47232 | 7,72E-110 | 76.0 |
| 50 | Singleton270 | 123 | CGMP-dependent protein kinase EGL-4 | *Ascaris suum* | ADY41468 | 0.0 | 91.0 |

*Values corresponding to the first singleton/contig in the list (with the higher number of reads)
