# Supplementary material for: The Transcriptome Analysis of Strongyloides stercoralis L3i Larvae Reveals Targets for Intervention in a Neglected Disease
Source: PLoS Negl Trop Dis. 2012 Feb 28;6(2):e1513. doi: 10.1371/journal.pntd.0001513 (PMC3289599; doi:10.1371/journal.pntd.0001513)
Supplement: Table S5 — S. stercoralis L3i putative proteins found similar to known therapeutic targets in parasitic nematodes either by protein domains mapping or sequence similarity. (DOCX) [file pntd.0001513.s006.docx]

**Table S5. *S. stercoralis* L3i putative proteins found similar to known therapeutic targets in parasitic nematodes either by protein domains mapping or sequence similarity.**

Tubulin β (IPR002453)

| Sequence ID | Similarity to *C. elegans* proteins (1E-15) | Similarity to human proteins (1E-05) | Secretory |
| --- | --- | --- | --- |
| Contig 498 | yes | yes | yes |
| Singleton 111 | yes | yes | yes |
| Singleton 1404 | yes | yes | yes |
| Singleton 5009 | yes | yes | yes |
| Singleton 8431 | yes | yes | yes |
| Singleton 8767 | yes | yes | yes |
| Singleton 9403 | yes | yes | yes |

γ-amino butyric acid A receptor (IPR006028)

| Sequence ID | Similarity to *C. elegans* proteins (1E-15) | Similarity to human proteins (1E-05) | Secretory |
| --- | --- | --- | --- |
| Contig 248 | yes | yes | no |
| Singleton 10259 | yes | yes | yes |
| Singleton 3637 | yes | yes | no |
| Singleton 3842 | yes | yes | yes |
| Singleton 4761 | yes | yes | no |
| Singleton 5144 | yes | yes | no |
| Singleton 6107 | yes | yes | no |
| Singleton 6757 | yes | yes | yes |

Glutathione S-transferase, C-terminal (IPR004046)

| Sequence ID | Similarity to *C. elegans* proteins (1E-15) | Similarity to human proteins (1E-05) | Secretory |
| --- | --- | --- | --- |
| Singleton 1058 | no | yes | no |
| Singleton 1590 | yes | yes | no |
| Singleton 3842 | yes | yes | yes |
| Singleton 4291 | yes | yes | no |
| Singleton 802 | yes | yes | no |

Glutamate-gated chloride channel (IPR015680)

| Sequence ID | Similarity to *C. elegans* proteins (1E-15) | Similarity to human proteins (1E-05) | Secretory |
| --- | --- | --- | --- |
| Contig 248 | yes | yes | no |
| Singleton 2729 | yes | yes | no |
| Singleton 3842 | yes | yes | yes |

Na-Asp-2 (*Ancylostoma* secreted protein-2)

| Sequence ID | Similarity to *C. elegans* proteins (1E-15) | Similarity to human proteins (1E-05) | Secretory |
| --- | --- | --- | --- |
| Contig 45 | no | yes | no |
| Singleton 56 | yes | yes | yes |
| Singleton 84 | no | yes | no |
| Singleton 97 | no | yes | no |
| Singleton 540 | no | yes | no |
| Singleton 684 | no | yes | no |
| Singleton 2713 | no | yes | no |
| Singleton 3096 | no | yes | no |
| Singleton 6827 | no | no | no |

Metalloprotease 1 precursor [*Ancylostoma caninum*]

| Sequence ID | Similarity to *C. elegans* proteins (1E-15) | Similarity to human proteins (1E-05) | Secretory |
| --- | --- | --- | --- |
| Contig 8 | no | no | yes (computationally predicted) |
| Singleton 4 | yes | yes | no |
| Singleton 159 | yes | yes | yes |
| Singleton 608 | yes | yes | yes |
| Singleton 993 | yes | yes | yes |
| Singleton 1562 | yes | yes | yes |
| Singleton 3610 | yes | yes | yes |
| Singleton 4900 | no | yes | no |
| Singleton 5895 | no | no | no |
| Singleton 10179 | yes | yes | no |

Cathepsin Z precursor [*Onchocerca volvulus*]

| Sequence ID | Similarity to *C. elegans* proteins (1E-15) | Similarity to human proteins (1E-05) | Secretory |
| --- | --- | --- | --- |
| Singleton 20 | yes | yes | yes |
| Singleton 387 | yes | yes | no |
| Singleton 836 | yes | yes | yes |
| Singleton 5330 | yes | yes | yes |
